# An Artificial Intelligence (AI)-Integrated Approach to Enhance Early Detection and Personalized Treatment Strategies in Lung Cancer Among Smokers: A Literature Review

**DOI:** 10.7759/cureus.66688

**Published:** 2024-08-12

**Authors:** Deep Chapla, Harshal P Chorya, Lyluma Ishfaq, Afrasayab Khan, Subrahmanyan VR, Sheenam Garg

**Affiliations:** 1 Medicine, Jiangsu University, Zhenjiang, CHN; 2 Internal Medicine, Baroda Medical College, Vadodara, IND; 3 Medicine, Directorate of Health Services Kashmir, Srinagar, IND; 4 Internal Medicine, Central Michigan University College of Medicine, Saginaw, USA; 5 Internal Medicine Pediatrics, Armed Forces Medical College, Pune, IND; 6 Medicine, Punjab Institute of Medical Sciences, Jalandhar, IND

**Keywords:** computational analysis, precision oncology, early identification, lung carcinoma, ai integration

## Abstract

Lung cancer (LC) is a significant global health issue, particularly among smokers, and is characterized by high rates of incidence and mortality. This comprehensive review offers detailed insights into the potential of artificial intelligence (AI) to revolutionize early detection and personalized treatment strategies for LC. By critically evaluating the limitations of conventional methodologies, we emphasize the innovative potential of AI-driven risk prediction models and imaging analyses to enhance diagnostic precision and improve patient outcomes. Our in-depth analysis of the current state of AI integration in LC care highlights the achievements and challenges encountered in real-world applications, thereby shedding light on practical implementation. Furthermore, we examined the profound implications of AI on treatment response, survival rates, and patient satisfaction, addressing ethical considerations to ensure responsible deployment. In the future, we will outline emerging technologies, anticipate potential barriers to their adoption, and identify areas for further research, emphasizing the importance of collaborative efforts to fully harness the transformative potential of AI in reshaping LC therapy. Ultimately, this review underscores the transformative impact of AI on LC care and advocates for a collective commitment to innovation, collaboration, and ethical stewardship in healthcare.

## Introduction and background

Cancer of the lung is the scariest disease on the planet. In 2020, the WHO reported that 1.8 million individuals lost their lives and 2.2 million new cases emerged due to this disease. In men, lung cancer (LC) is at the top of all cancers regarding morbidity and mortality; in women, it ranks third in terms of mortality after breast and colorectal cancer [1]. According to projections by the International Agency for Research on Cancer, the number of cases is expected to reach 2.5 million by the year 2025 and 3.5 million by the year 2040 [2]. LC is generally classified into two primary subcategories: non-small cell LC (NSCLC) and small cell LC (SCLC). Approximately 80-85% of lung malignancies are NSCLC, while SCLC constitutes 15-20%. The predominant forms of NSCLC include adenocarcinomas, squamous cell carcinomas, and large cell carcinomas. In contrast, adenosquamous carcinoma and sarcomatoid carcinoma are less frequently diagnosed [3].

One of the biggest risk factors for LC is smoking, especially cigarette smoking, which includes over 70 carcinogens [4]. Smoking can also affect LC prognosis, with people who have never smoked having a good prognosis, current smokers having a poor prognosis, and former smokers who quit a few years ago having a mixed prognosis [5]. Apart from smoking, primarily a few other factors, including nutritional factors, contribute to a bad prognosis: individuals with LC in stage I who possess a BMI below 18.5 kg/m are at an increased risk of premature mortality when compared to those with a normal BMI. The prognosis of NSCLC surgery is more favorable in females than in males. Other comorbidities, including diabetes, chronic obstructive pulmonary disease, coronary artery disease, and other primary cancers, contribute to an adverse prognosis [6].

**Figure 1:**
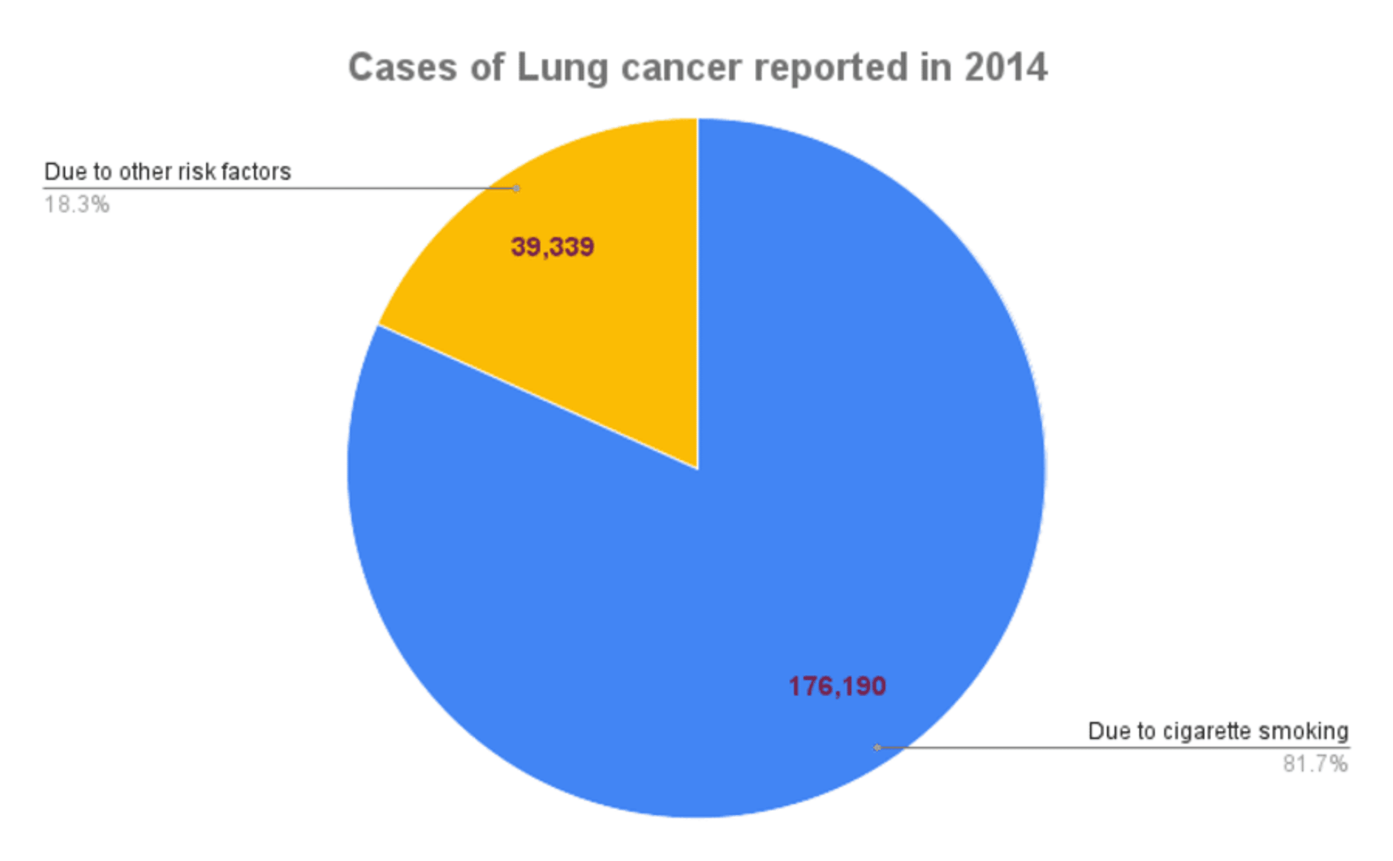
Reported cases of LC due to cigarette smoking in the United States in 2014 LC, lung cancer

**Figure 2:**
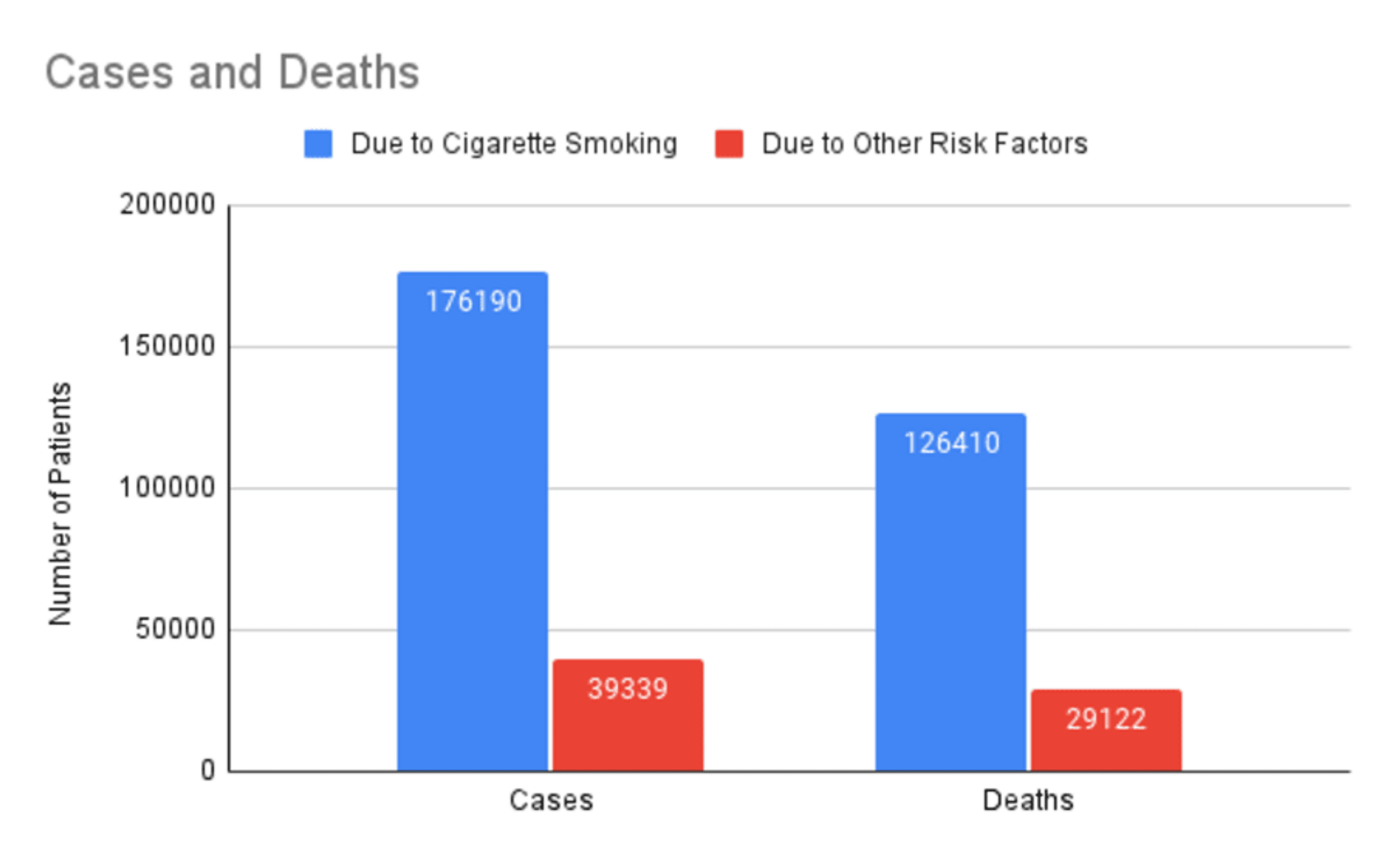
Comparison of new cases and deaths due to cigarette smoking versus non-smoking causes

In 2014, 176,190 newly diagnosed cases of LC were attributed to cigarette smoking as a risk factor. Of these, 95,180 were male and 81,010 were female. Smoking contributed to 81.3% (126,410) of LC fatalities in the same year. These data have been plotted on a pie chart (Figure 1) and a column chart (Figure 2) [7].

By offering early treatment, early cancer detection decreases morbidity and mortality in the population. A screening program for cervical cancer, breast cancer, and colon cancer exists in the United Kingdom; this program may relieve strain on the healthcare system [8]. Early detection of colorectal cancer is associated with an approximate 90% five-year survival rate [9]. Mammography screening can reduce breast cancer-related mortality by 28-45% [10]. Detection of LC early reduces 10-year mortality by 24% in males and 33% in females. In 2017, UK studies found that if LC was identified in the early stage (stage I), the one-year survival rate was 81-85%, whereas if it was detected in the late stage (stage IV), it was 15-19% [11]. According to these findings, the diagnosis of LC at an early stage is essential for lowering mortality rates.

LC is difficult to detect on chest radiography (CXR) because early-stage nodules are tiny and blood veins and ribs overlap with the cancer mass or nodules. CT scans are becoming more common for lung pathology identification, and multiple studies have shown that low-dose CT (LDCT) scans are optimal for lung nodule diagnosis [12]. Sputum analysis was a noninvasive screening for cancer a few years ago. On the other hand, randomized control trials conducted in the early 1970s at the Mayo Clinic, Johns Hopkins University, and Memorial Sloan Kettering Cancer Center demonstrated that sputum cytology did not serve as a screening method for LC [13].

As we addressed the benefits of early detection to minimize mortality and improve treatment outcomes, screening of high-risk individuals should be performed, which increases radiologists’ workload [14]. There are no criteria to help pulmonologists and radiologists classify tiny nodules as benign or malignant. Therefore, numerous CT scans and biopsies are required, which is stressful [15]. The field of artificial intelligence (AI) has experienced significant growth over the past few decades, resulting in a multitude of implications across various domains. Furthermore, it has significantly contributed to the healthcare sector by performing tasks such as data analysis, diagnosis, treatment plan formulation, and disease prognosis verification [16].

New inventions have occurred in all disciplines. The healthcare industry is witnessing an expansion of technologies that have supplanted several conventional approaches. Robotic surgery and laparoscopic surgery, which are less invasive techniques, have significantly replaced open surgeries. Telemedicine has replaced conventional consultations since the COVID-19 pandemic and has been beneficial for those who reside in remote areas. Advanced imaging techniques improve prognosis by facilitating the early detection of certain terrifying diseases [17].

John McCarthy coined the term AI in 1955 [18]. Since then, a great deal of research has been conducted on the subject, and in the last 20 years, AI has become increasingly prevalent in everyday life [19]. In the current decade, AI is the most popular technology. It exerts diverse influences across multiple domains, including information and technology, marketing, healthcare, cybersecurity, the arts, and the military.

This article explores the potential utility of various AI approaches in identifying individuals at risk of developing LC, performing screening and diagnostic procedures, and developing treatment strategies. Researchers have looked at electronic medical records (EMRs) using the Extreme Gradient Boosting (XGBoost) machine learning (ML) algorithm to predict how likely it was that someone would get LC within a year [20]. The article discusses several significant studies that have leveraged advanced technologies in LC prediction and treatment. One study utilized deep learning and convolutional neural networks (CNNs) to accurately predict one-year LC rates based on clinical data from EMRs [21]. Another study employed computer-aided detection (CADe) to identify lung nodules on CT scans, categorizing them by various factors such as size, shape, and density, using CNNs [22]. Radiomics, on the other hand, plays a crucial role in distinguishing between different types of LC and predicting recurrence post-resection [23,24]. Moreover, radiomics aids in evaluating the effectiveness of chemoradiation in LC treatment [25]. The insights gained from these studies contribute to the development of datasets for future AI applications, with paramount considerations regarding patient autonomy and confidentiality [22].

Although AI technology is extremely beneficial to healthcare, it faces several obstacles. There are currently no large data sets or datasets available for training, validating, and testing models [26]. When it comes to the development of AI in the future, ethical concerns will be of the utmost importance because permission and ethical norms will be adopted in order to create further data sets from EMR [27].

Furthermore, we discuss personalized treatment strategies, early detection of LC, and the current state of AI integration in LC, as well as prospective directions and challenges, in this review.

## Review

Early detection of LC

Traditional Approaches and Limitations

LC continues to be a major worldwide health concern, with the greatest number of cancer-related fatalities observed in both males and females. With approximately 1.8 million new cases and 1.6 million fatalities annually worldwide, LC constitutes approximately 23% of cancer-related deaths in the United States alone [28,29]. The five-year survival rate, currently at only 20.5%, underscores the challenge, often exacerbated by late-stage diagnoses at initial presentation. At present, the diagnosis of LC is primarily based on clinical evaluation when patients exhibit symptoms such as persistent coughing, discomfort, and weight loss. Unfortunately, individuals experiencing these symptoms typically develop advanced-stage LC. Timely identification is crucial, as the mortality rate for LCs is much reduced in the early stages when they can potentially be cured with surgical excision [29].

In the 1980s, CXR and sputum cytology were studied as screening tools for LC. However, they did not notably reduce LC mortality and were associated with drawbacks such as length bias and overdiagnosis. The Prostate, Lung, Colorectal, and Ovarian (PLCO) Cancer Screening Trial, which involved annual CXR screening for four years in the intervention group compared to routine care in the control group, found no significant mortality benefit after 13 years of follow-up (RR: 0.99; 95% CI: 0.87-1.22) [28,30].

In the 2000s, studies assessed the use of LDCT for LC screening. Although CT scans identified more cases of early-stage cancer, there was initially inconclusive evidence of a mortality benefit. The National Lung Screening Trial (NLST) conducted in 2011 was a significant study that showed a 20% reduction in mortality when utilizing LDCT compared to chest X-rays (CXR) in a group at high risk for LC. This research led to an increase in the acceptability of LDCT for LC screening [31]. From August 2002 to April 2004, more than 50,000 adults at high risk for LC were randomly divided into two groups. One group underwent three annual tests using LDCT, while the other group underwent screenings using CXR. The follow-up period was extended to December 31, 2009. The study found that over 90% of the participants adhered to screening. The positive screening rates were 24.2% for LDCT and 6.9% for radiography throughout all three rounds, with the majority of false positives (96.4% for LDCT and 94.5% for radiography). LC was more prevalent in the LDCT group than in the radiographic group. LDCT screening resulted in a substantial decrease in LC mortality, demonstrating a relative reduction of 20.0% compared with radiography. The LDCT group exhibited a 6.7% reduction in overall mortality. Trials such as the NELSON and the MILD supported LDCT screening by providing additional evidence of mortality reduction after a 10-year follow-up [31].

LDCT is a scanning technique designed to minimize the radiation dose received by patients during imaging procedures while maintaining the quality of diagnostic images. It typically involves using a tube current of less than 100 mA, with common settings ranging between 40 and 80 mA. This approach is complemented by adjusting the tube voltage, which is usually set at approximately 120 kV and may vary according to the patient’s weight. For optimal imaging, different slice thicknesses were used, depending on the focus of the scan. For non-targeted fields of view in low-frequency algorithms, a slice thickness of 2.5 mm or higher was maintained. In contrast, for targeted areas or regions of interest, a thinner slice thickness (1.0-1.5 mm) is used with high-frequency algorithmic reconstruction to enhance spatial resolution. The radiation dose from LDCT varies depending on the combination of kVp and mAs used, but on average, an effective dose of around 2 mSv is considered acceptable for LDCT screening [32].

In 2013, the United States Preventive Services Task Force (USPSTF) recommended that individuals at high risk for LC undergo LDCT screening. As of the latest update, the USPSTF recommendation is that individuals aged 50-80 who have a smoking history equivalent to smoking 20 packs of cigarettes per year and who currently smoke or have quit within the past 15 years should undergo annual LC screening using LDCT [33]. In 2015, the Centers for Medicare and Medicaid Services granted approval for the use of LDCT as a screening method for people at high risk. This approval came with certain requirements, including counseling and shared decision-making (SDM) consultations [28].

Nevertheless, the existing screening criteria fail to sufficiently account for variations in race, ethnicity, socioeconomic position, sex, specific comorbidities, and healthcare accessibility. Existing criteria might not adequately account for groups such as African-Americans, who have a higher risk of LC at a younger age, and women, who have a higher risk regardless of differences in smoking habits [34]. Other constraints include the occurrence of both false positives and false negatives. Raising the threshold for nodule detection can lower the number of false positives but may also result in a decrease in sensitivity. Observer errors, lesion characteristics, or technical errors may lead to false negatives. Overdiagnosis can result in unnecessary medical interventions and exacerbate morbidity, anxiety, and financial burdens. The probability of any LC identified with LDCT screening being an overdiagnosis is 18.5% (95% CI: 5.4-30.6%) [35].

In the NLST, over 90% of screened nodules were not malignant [36]. [Current guidelines do not adequately assist in distinguishing benign from malignant small indeterminate nodules, leading to close monitoring through serial CT scans and sometimes biopsies. These procedures can cause patient anxiety and carry risks, especially biopsies, which are invasive and have the potential for morbidity. The psychosocial impact is an additional concern. Screening may lead to the development of anxiety, depression, and mental distress. There is also a lack of clear guidance on when to intervene while monitoring the nodules. Although the American College of Radiology’s Lung-RADS offers some recommendations, it involves a subjective interpretation. In this scenario, AI could play a crucial role in more accurately identifying nodules that require intervention, thereby optimizing both the initial screening and follow-up processes [28].

Additionally, there is a potential for cancer development due to exposure to ionizing radiation. Risk accumulates over an individual’s life span. For individuals between the ages of 50 and 75 who smoke and undergo LDCT screening, annual screening is projected to increase the baseline risk of cancer in males (15.8%) by 0.23% and in females (16.9%) by 0.85%. This represents a 1.5% increase in risk for males and a 5% increase in risk for females [37].

Role of AI in Risk Prediction Models

The integration of AI into the LC screening process represents a significant advancement in the field of oncology. It improves the precision and effectiveness of screening and facilitates the development of more personalized and targeted methods for detecting and treating cancer. The advancement of AI technology is anticipated to further enhance its significance in early LC diagnosis and risk prediction models, potentially resulting in enhanced survival rates and improved patient outcomes.

Risk prediction is more significant in LC screening than clinical assessment, as evidenced by several trials, including the PLCOm2012 [37] and Liverpool Lung Project models [38]. One risk stratification approach for LC screening programs could be to use risk prediction models to identify screening candidates. CT screening data can be used to establish personalized screening intervals for individuals.

AI Applications in EMR

The integration of AI into the management of LC is progressively utilizing data obtained from EMRs. EMRs are of paramount significance because they include extensive patient care data, including imaging, pathology, and treatment information.

Wang et al. aimed to develop and validate a risk prediction model for identifying patients at risk of developing LC within the following year in the general population. Using data from electronic health records (EHRs) in the Maine Health Information Exchange network, this study involved two cohorts: a retrospective cohort for model construction and a prospective cohort for validation. The model utilized the XGBoost algorithm to analyze clinical profiles spanning six months, assigning a risk score for potential new LC occurrences. With a notable area under the curve (AUC) of 0.881 in the prospective cohort, the model exhibited significant accuracy. Categorizing the population into low-, medium-, and high-risk groups based on two score thresholds, the study revealed that the high-risk category had an LC incidence rate 7.7 times higher than the overall cohort. Key predictive factors for new LC cases include age, prior pulmonary and chronic diseases, medications for mental disorders, and socioeconomic disparities. However, it is important to note that the study lacks a comparison with standard diagnostic methods involving clinical evaluation and imaging [20]. Further evaluation is needed to ascertain whether the model indeed aids in improving prognosis and treatment outcomes for patients. Nonetheless, the study underscores the efficacy of leveraging EHR data statewide to accurately identify individuals at a high risk of developing LC within a year.

Kehl et al. focused on developing a deep neural network natural language processing (NLP) model to predict the prognosis and treatment needs of patients with solid tumors or lymphoma. This algorithm, which was trained using 302,688 imaging reports, was aimed at predicting the probability of patients requiring new palliative-intent systemic therapy within 30 days. The model’s performance, evaluated using Harrell’s C-index and the area under the receiver operating characteristic (ROC) curve, showed strong predictive ability, achieving a c-index of 0.76 for survival prediction and an AUC of 0.77 for initiating new treatment. Furthermore, AUC values of 0.78 and 0.84, showed a significant correlation between the model’s outputs and manual annotations of cancer progression. The study found that deep NLP models can predict clinical inflection points in cancer patients and enable real-time, targeted clinical trial screening treatments on a health system scale, improving patient care [39].

Utilization of AI in LDCT Imaging

The utilization of AI-based reconstruction approaches in LDCT imaging allows for a greater decrease in the radiation dose while retaining excellent image quality and limiting the potential dangers associated with radiation exposure during screening. ML can examine large amounts of imaging and clinical data to customize screening regimens. This risk stratification aids in the identification of individuals with an elevated risk of acquiring LC, thereby maximizing the allocation of screening resources and directing efforts toward those who require it the most. AI-driven computer-aided design (CAD) systems can automatically identify possible lung nodules with a high level of sensitivity. These devices can operate as either concurrent or secondary readers, thereby substantially decreasing the duration needed for radiologists to analyze imaging findings. The exceptional sensitivity of AI in identifying lung nodules guarantees a reduced number of overlooked cases, hence enhancing the likelihood of early identification [40].

Advanced technologies such as MRI, CT, and PET play a crucial role in collecting detailed data for LC analysis. These imaging techniques provide a wealth of data that can be thoroughly analyzed using radiomics, CAD, and CNNs. By incorporating this imaging data with other crucial factors such as gene expressions, mutations, and molecular signatures, healthcare professionals can attain a more precise and reliable prognosis [22].

Deep learning techniques, such as CNNs, can be used to identify visual patterns in raw pixel data using transformations and filters. They have been effectively used to classify nodules during LC screening using LDCT scans. Paul et al. employed a combination of custom-trained CNNs, pretrained CNNs, and radiomic characteristics to improve the accuracy of nodule malignancy prediction in LC screening. The efficacy of this collective methodology was evaluated using subsets of participants from the NSLT. Their approach yielded an accuracy rate of 89.45% and an AUC value of 0.96 [41].

Ardila et al. analyzed LDCT scans from the NLST and created a deep-learning algorithm designed to evaluate the risk of LC using both the latest and previous CT scans. This model demonstrated outstanding performance, with an AUC of 94.4%. Two reader studies were included to compare the performance of the model with that of human radiologists. In situations where prior CT images were not available, the algorithm surpassed the performance of the six radiologists, reducing false positives by 11% and false negatives by 5%. However, when prior CT images were available, the model’s performance was comparable to that of the radiologists [42].

Chen et al. focused on differentiating between SCLC and NSCLC adenocarcinoma using CT radiomics, a challenging method because of the subtle visual differences in conventional imaging. The study involved patients with primary LC (SCLC or NSCLC adenocarcinoma) using pretreatment CT images. Radiomic features were extracted using histogram-based statistics, textural analysis, and the wavelet transform of tumor images. A multilayer artificial neural network (ANN) was employed to construct the predictive model with feature selection aided by a minimal-redundancy-maximal-relevance method. The developed SCLC/NSCLC classifier achieved an impressive AUC of 0.93, with a sensitivity and specificity of 0.85. It was observed that incorporating clinical data, such as smoking history, could slightly enhance model performance. Textural features emerged as the top-ranking radiomic features, indicating that CT radiomics can effectively represent tumor heterogeneity and distinguish between LC subtypes. Wavelet transformation techniques further enhanced the radiomic features of the SCLC/NSCLC classification. This indicates the promise of radiomics as a noninvasive instrument for early detection and treatment planning [22,23].

AI-Based Imaging Analysis for Early Diagnosis

LC is mainly classified based on histology into NSCLC and SCLC. NSCLC includes various subtypes, such as adenocarcinoma, squamous cell carcinoma, large cell carcinoma, and squamous adenocarcinoma. The International Association for Lung Cancer Research classifies LC into stages I-IV, with stages I and II as early stages and stages III and IV as advanced LC. The staging criteria were based on the tumor diameter, lymph node metastasis, and distant metastases.

CT Datasets for LC

The Lung Image Database Consortium and Image Database Resource Initiative (LIDC-IDRI) is the most widely used CT dataset in LC research. In 2000, the US National Cancer Institute began creating a CT-based lung nodule reference database, which was eventually supplemented with private company CT images with support from the Foundation for the National Institutes of Health [43]. Another significant dataset is the Lung Nodule Analysis 2016 (LUNA16), established as a challenge for the computer science community to develop effective computer-aided diagnostic models for lung nodule detection. The LUNA16 dataset is a high-quality subset of the LIDC-IDRI, excluding CT scans with a slice thickness greater than 2.5 mm [44]. Additional noteworthy datasets include the Aliyun Tianchi dataset and a large-scale CT and PET/CT dataset from the Computer Center and Cancer Institute at the Second Affiliated Hospital of Harbin Medical University in China. These datasets have been extensively used in the past decade for lung nodule analysis and have significantly contributed to advancements in this field.

In the field of AI-assisted image analysis, CAD systems are mainly classified into two categories: CADe and computer-aided diagnosis (CADx) systems. CADe is a specialized system that aims to identify and pinpoint the presence of abnormalities in the lungs, serving as a vital tool in the early identification process. In contrast, CADx takes an additional step by precisely characterizing these abnormalities, including the crucial task of distinguishing between malignant and benign ones [45]. Multiple of these pioneering CAD systems have obtained endorsement from the FDA [46]. It is crucial to acknowledge that these systems are primarily intended to emphasize potential areas of interest. Their classification of nodules is not definitive in determining whether they are benign or malignant; instead, it assists clinicians in identifying areas that may necessitate additional investigation.

A CAD system for LC diagnosis based on CT images typically encompasses three primary stages: data loading and preprocessing, lung nodule detection, and lung nodule classification. AI facilitates this process using two primary methodologies: automatic segmentation and assessment of detected nodules, along with radiomic feature extraction for virtual biopsy.

Data Loading and Preprocessing

The initial stage of data loading and preprocessing involves gathering CT scans and pertinent patient metadata. Subsequently, preprocessing techniques were employed to reduce technical noise and discrepancies. Segmentation techniques are utilized to precisely separate lung tissue from other components such as adipose tissue, air, and osseous tissue. Segmentation can be accomplished using simple value thresholding-based techniques or more advanced deep learning algorithms, such as CNNs, U-Net, and generative adversarial networks (GANs). Particular emphasis is placed on LDCT scans, which pose challenges because of their lower resolution and signal-to-noise ratios. Complex deep learning-based image denoising methods, such as GAN-based super-resolution approaches, are used to improve image quality. These methods provide accurate and reliable information for further diagnostic analysis.

Makaju et al. proposed a novel approach that integrates image processing and ML to improve the accuracy of LC identification from CT images. Instead of using the Gabor filter during the preprocessing phase, this new model uses Gaussian and median filters. After preprocessing, the images were subjected to watershed segmentation, which emphasizes the cancer nodules. The model has a cancer detection accuracy of 92%, while the classifier achieves an accuracy of 86.6% [47].

Lung Nodule Detection

CNNs are highly effective in detecting lung nodules on CT scans, making them the next crucial stage in the process. Important progress has been made in the development of three-dimensional deep CNN models that incorporate multi-scale prediction. These models successfully leverage spatial and contextual information from CT scans to improve nodule detection accuracy. The integration of techniques such as the attention mechanism aims to mimic the detection patterns of radiologists, thereby improving the concentration of pertinent regions in the scans. For example, the incorporation of attention modules in U-Net and the utilization of group-attention single-shot detection have demonstrated a high level of sensitivity in detecting pulmonary nodules while simultaneously maintaining a low percentage of false-positive results [48,49]. The three-dimensional (3D) multi-conditional GAN technique is capable of producing realistic synthetic lung nodules, thereby improving the diversity and volume of training data [50].

Zhao et al. conducted a study in which they created a deep learning system that utilized 3D CNNs and multitask learning. The purpose of this system is to predict the invasiveness of tumors and build 3D nodule segmentation masks from CT scans. This study focused on early-stage lung adenocarcinoma. The deep learning system demonstrated superior performance compared to the radiologists, getting an F1 score of 63.3%, while the radiologists’ results ranged from 51.0% to 56.6% [51]. In another study, Dobbins III developed an automated method for lung segmentation to detect lung nodules in digital chest tomography, achieved relatively high accuracy for lung segmentation, and accurately recognized all lesions [52].

Khosravan and Bagci introduced a novel deep learning method, S4ND, to detect lung nodules on CT scans. This method significantly improves computational efficiency compared to existing multi-stage frameworks. S4ND utilizes a single 3D CNN with dense connections, operating in a single feed-forward pass, eliminating the need for multiple stages in the detection process. The network was trained end-to-end without requiring additional postprocessing or user intervention to refine the detection results. When tested on the LUNA challenge dataset, it achieved an average FROC score of 0.897 [53].

Optical coherence tomography is an advanced imaging technology that utilizes low-coherence light to provide greater spatial resolution than CT. It can produce high-definition cross-sectional images of the inner lungs, including the luminal wall of the airway. This technique can be utilized to identify any abnormalities in the bronchial region, which can be indicative of the presence of LC [13].

Lung Nodule Classification

After the identification of lung nodules using CT scans, the next crucial task was to categorize them as either benign or malignant. Classifying lung nodules as benign or malignant is a challenging task, particularly because of the visual similarities between the two types. Traditional ML methods apply non-deep-learning algorithms such as random forests and support vector machines (SVMs) for classification but fall short in robustness and flexibility, particularly when differences between nodules are subtle.

Deep learning-based methods, particularly CNNs, offer a data-driven approach to nodule classification. Both 2D and 3D CNNs have shown reasonable effectiveness in lung nodule classification. Advanced methods such as multi-scale CNNs use classifiers such as SVM and random forest for final nodule classification. Setio et al. introduced a multiview 2D CNN method that incorporates spatial contextual information from different perspectives to improve the representation of lung nodule features [49,54].

Nasrullah et al. utilized two 3D deep CNNs, named CMixNet, to perform lung nodule identification and classification. The detection process utilizes a combination of a faster R-CNN, CMixNet-trained features, and a U-Net-like architecture. In contrast, classification is performed using a gradient-boosting machine on the features learned from CMixNet. This study emphasizes the significance of the IoT and wireless body area networks in the continuous monitoring of patients, which assists in the early detection of LC. The integration of deep learning and clinical methodology yielded remarkable outcomes on the LIDC-IDRI datasets, demonstrating a sensitivity of 94% and a specificity of 91%. This surpassed the performance of previous techniques [55].

The study conducted by Al-Shabi et al. employed a novel CNN architecture called gated dilated (GD) networks to accurately categorize lung nodules as malignant or benign. GD networks use multiple dilated convolutions to successfully capture variations in nodule size. GD contains a specific subnetwork that examines input features and guides them to appropriate dilated convolutions, thereby improving the network’s flexibility and precision. After conducting tests on the LIDC-LDRI dataset, the GD network exhibited exceptional performance, with an AUC value of 0.95. Significantly, the GD network demonstrated improved accuracy in classifying nodules of medium size compared to the baseline models [56].

Sibille et al. conducted a retrospective study to assess the effectiveness of deep CNNs in accurately identifying and categorizing uptake patterns in whole-body 18F-FDG PET/CT images for patients diagnosed with LC and lymphoma. Nuclear medicine professionals carefully identified and classified regions with higher 18F-FDG uptake as either suggestive or non-suggestive of cancer. Subsequently, a CNN was developed to detect these areas, predict their anatomical locations, and classify them according to expert assessments. The CNN exhibited exceptional accuracy, as indicated by the remarkably high AUCs for the test set. When merging 18F-FDG PET and CT information, the AUC ranged from 0.98 to 0.99. The CNN demonstrated exceptional precision in anatomical localization, accurately identifying the specific body part, region, and subregion in a significant majority of cases [57].

Personalized treatment strategies

Challenges in Conventional Treatment Approaches

The primary modalities for the treatment of LC include surgical resection, radiotherapy, and pharmacotherapy. Based on staging, tumor location, and histological and genetic alterations, a range of treatment strategies are available, allowing for the selection of the most optimal treatment. Drug therapies for LC include chemotherapy, targeted therapy, and immunotherapy. The selection of optimal therapeutic drugs for personalized medicine remains an unresolved issue. The surgical treatment options for LC include wedge resection, segmental resection, lobectomy, and pneumonectomy. The majority of tumor patients present with inoperable tumor lesions and concomitant severe medical conditions such as hypertension, heart disease, and diabetes, among others, leading to a diminished quality of life following surgical intervention [58]. The five-year survival rate following surgical intervention remained at approximately 50% [59].

Although there have been significant breakthroughs in the treatment of NSCLC, it remains difficult to accurately assess treatment outcomes and detect disease progression. Most clinical trials use established criteria, like the Response Evaluation Criteria in Solid Tumors (RECIST) and the WHO guidelines, to judge how well a tumor has responded. Nevertheless, the emergence of immunotherapy has led to the increasing acceptance and use of immune-related RECIST criteria. Although these criteria are useful, they can sometimes fail to accurately correlate with real treatment responses, mistakenly interpreting posttreatment changes, such as inflammation and pneumonitis, as tumor progression. AI, specifically in the form of radiomics, has the potential to greatly improve the ability to differentiate between changes caused by treatment and actual disease progression [22].

A study conducted by Mattonen et al. utilized CT texture analysis after stereotactic body radiation therapy to distinguish between recurrence and radiation-induced lung injury (RILI). This study examined a dataset consisting of 13 lesions with moderate to severe RILI and 11 lesions with recurrence. This revealed the shortcomings of the RECIST, which achieved an accuracy rate of only 65.2%. The RECIST method showed significant rates of false-negative and false-positive results. Conversely, the utilization of radiomics greatly enhanced the precision to 77%, showcasing an AUC of 0.81 [22,60].

AI-Driven Biomarker Identification

Modern pathological evaluations of LC involve a thorough investigation that looks at many factors, such as the complex features of the tumor’s environment, the presence of biomarkers that are important for choosing the right treatments and predicting response (e.g., PD-L1), and a full analysis of the tumor's genomic profile [57]. The predominant biomarkers for predicting LC include Rb, K-RAS, EGFR, c-MET, TP53, ALK, and PDL1. Despite the identification of multiple potential biomarkers, their clinical usefulness is limited by the inconsistent nature of diagnosis and prognosis prediction. Currently, AI-facilitated proteomics is experimenting with multiple biomarker panels to enhance the detection of various forms of LC and aid in the selection of optimal therapeutic approaches based on individual tumor characteristics [61].

The utilization of CNNs for tumor characterization has become more widespread. According to a study by Coudray et al., CNNs are very effective in differentiating between non-malignant, adenocarcinoma, and squamous cell carcinoma. This study utilized an analysis of the spatial distribution of cancer cells in section images to predict the ten most frequently mutated genes in adenocarcinoma. Of these 10 genes, six (STK11, EGFR, FAT1, SETBP1, KRAS, and TP53) were accurately predicted, with a success rate ranging from 73.3% to 85.6% using pathological images. In addition, a conditional generation antagonistic network was employed to segment nuclei in cancerous epithelial tissues. Additionally, the accuracy of predicting the risk of EGFR gene mutation was enhanced by incorporating effective histopathological features and utilizing a SVM classifier. The results suggest that AI has the potential to greatly improve the speed and accuracy of diagnosing LC [22,58,62].

Another study conducted by Wang et al. employed CNNs, particularly a model called Conf Path, to categorize cell types present in the tumor microenvironment. The ConvPath software demonstrated notable classification accuracy, with 92.9% during training and 90.1% during testing on the datasets. Additionally, it can transform pathological images into spatial maps that identify various cell types. Subsequently, these maps were used to construct a prognostic model based on image features, which was verified in two separate cohorts. This model can autonomously predict risk groups for LC prognosis by taking into account factors such as age, gender, smoking status, and stage [63].

Immunohistochemistry plays a vital role in subtyping NSCLC using molecular markers. However, conducting multiple stains on a limited number of tissue samples is a significant challenge. Koh et al. tackled this issue by utilizing decision trees and SVM ML classifiers, specifically in situations with uncertain outcomes. Their approach exhibited an accuracy ranging from 72.2% to 91.7%, depending on the marker pattern. However, it offered a practical methodology for subtyping small NSCLC biopsies using a minimal panel of three markers [64].

One major advancement in cancer diagnostics is the introduction of liquid biopsies, which are particularly useful for capturing temporal heterogeneity. Zhang et al. conducted a noteworthy study on synthetic minority oversampling with random forests using an innovative approach. This method, applied in conjunction with random forests, was used to identify LC through circulating miRNA, achieving an impressive AUC of 0.99 [65].

ML techniques play an increasingly pivotal role in the evolving landscape of cancer genomics, particularly in the analysis of gene expression profiles. Moses et al.’s work, which used a novel ML algorithm to subclassify lung squamous cell carcinoma and lung adenocarcinoma, is a prime example of this application. By identifying previously unknown mutations, such as PIGX, an oncogenic driver in breast cancer, this study opens new avenues for research and potential therapeutic interventions. Unlike supervised learning, unsupervised learning ML algorithms that are crucial to this research can find data patterns and commonalities without labels. Gene expression analysis benefits from this approach because it allows the discovery of novel mutations and genetic variations without predetermined categories [22,66].

Daneshkhah et al. conducted a study to explore the use of chromatin-sensitive partial wave spectroscopic microscopy (csPWS) combined with AI for the noninvasive detection of early-stage LC. Previous research has indicated that cells in the buccal mucosa of smokers might show molecular changes indicative of LC, a concept known as field carcinogenesis. This study developed a method called csPWS to identify changes in the structure of chromatin in the buccal mucosa that can serve as a biomarker for LC. The effectiveness of the AI-enhanced csPWS technique was assessed in 179 patients from two different clinical locations. The test exhibited exceptional accuracy in distinguishing stage I LC from individuals without cancer, achieving an area under the receiver operating characteristic curve of 0.92 at Site 1 and 0.82 at Site 2. A simple, self-administered buccal swab was analyzed in a central laboratory. Implementing this strategy can potentially enhance patient outcomes by substantially augmenting the efficacy and availability of LC screening. The study’s limitations are its small sample size and the fact that the majority of participants are smokers [67].

Tailoring Therapeutic Interventions Using AI Algorithms

Treatment for LC encompasses a range of options, including surgical and nonsurgical approaches such as immunotherapy, radiation therapy, and chemotherapy. Radiotherapy plays a vital role in LC treatment. Recent advancements in AI have shown significant potential for enhancing the efficacy of radiotherapy. ML systems are being more and more utilized to exploit the extensive volume of data produced by radiotherapy procedures. The dataset included CT scans and detailed treatment records. By analyzing these data, ML algorithms can be employed to optimize the angles of radiation beams, thereby achieving the highest therapeutic efficacy while minimizing potential damage to adjacent healthy tissues. Furthermore, these algorithms exhibit expertise in predicting dose-volume histograms, which are crucial for assessing the radiation dose received by both the tumor and adjacent healthy tissues [61].

AI plays a crucial role in making treatment decisions by utilizing clinical decision support systems such as IBM Watson for Oncology (WFO) to predict surgical risks and make treatment decisions. WFO facilitates the extraction of crucial information from medical records, the presentation of pertinent evidence, and the identification of potential treatment alternatives. A comparative analysis between decisions made by the WFO and a multidisciplinary team revealed a significant level of concordance in recommendations for early-stage and metastatic disease, ranging from 92.4% to 100%. However, the concordance rates were comparatively lower for stage II or III, ranging from 80.8% to 84.6% [66,68].

The utilization of WFO in nations such as China and Korea has shown great potential, particularly in delivering precise treatment suggestions for patients with advanced-stage LC. The system’s recommendations were in accordance with those of the multidisciplinary teams. However, challenges arise because of regional variations in genetic mutations, treatment protocols, drug availability, and coexisting diseases, including differences between the United States and other countries. Optimizing the effectiveness of WFO is crucial by tailoring it to regional and individual patient factors [61].

AI has also made notable advancements in pharmaceutical research and development. A prime example of this can be seen in the study conducted by Li et al., who employed an innovative AI-driven methodology for drug repurposing. This methodology integrates deep learning with transcriptomic data and chemical structure analysis. By employing their inventive approach, they recognized pimozide, a preexisting medication utilized for Tourette's disorder, as a highly promising option for treating NSCLC. The efficacy of pimozide in this novel capacity was confirmed by its ability to induce cell death in A549 cell lines, a specific type of LC cells, thereby showcasing the potential of AI in discovering novel uses for established drugs in the field of oncology [22,69].

Pei et al.’s study emphasizes the utilization of AI to customize LC treatment, specifically in the process of choosing appropriate therapeutic medications. They proposed an integrated learning collaborative filtering method in the field of ML. This method has two primary purposes. First, it simplifies the decision-making process to choose the most efficient compounds, thus enabling personalized drug therapy for LC. Additionally, it assists in the identification of potential drug targets and candidates, thereby facilitating the advancement of personalized medications [58].

In addition, the QUANIC system employs an ML technique for baseline covariates. This approach uses distinctive, extensive, multimodal, and longitudinal data acquired from groundbreaking clinical studies. These models primarily aim to comprehend and forecast reactions and resistance to immunosuppression at the immune checkpoints. The ultimate objective is to attain individualized immunotherapy for patients with LC [58].

AI tools are currently being actively developed to predict responses to immune checkpoint inhibitors and targeted therapies for the treatment of LC. Charoentong et al. performed extensive immunogenomic ML analysis on multiple types of cancer, including LC, to forecast the effectiveness of checkpoint inhibitors. As a result, an “immunophenoscore” was developed, which was more accurate than PD-L1 expression in predicting the effectiveness of immunotherapies for specific cancer types. Subsequently, they devised a scoring system named the “immunophenoscore.” The scoring system was determined to be a more accurate indicator of how well patients would respond to anti-cytotoxic T lymphocyte antigen-4 and anti-programmed cell death protein 1 antibodies in the two separate validation groups. The discovery and creation of this resource are anticipated to have a substantial impact on cancer immunotherapy and the progress of precision immuno-oncology [22,70].

Kureshi et al. utilized ML techniques, specifically an SVM and decision tree classifiers, to assess various factors in forecasting tumor response in patients with EGFR-positive NSCLC who were administered erlotinib or gefitinib. The data-driven decision support model demonstrated a predictive accuracy of 76% and an AUC of 0.76 [22,61,71].

Current state of AI integration in LC care

Review of Existing Studies and Clinical Trials

Early detection and prompt treatment of LC are challenging, even with current technology. AI algorithms such as ML, deep learning, and radiomics can detect, screen, and help differentiate between benign and malignant lung nodules [22]. This, in conjunction with clinical data, imaging data, biomarkers, and tumor markers, can be used in comprehensive diagnostic assessment, LC staging, treatment response prediction, and therapy optimization [72].

With the advent of improved algorithms, computational power has increased, and the increased availability of big data in a streamlined manner has driven the use of AI in clinical oncology. Data from EMR systems are being used in this regard. One such is XGBoost by Ladbury et al., an ML algorithm that yielded an AUC of 0.88. Another NLP model to predict one-year LC risk in patients yielded an AUC of 0.77 [22].

The NLST has shown that LDCT resulted in a 20% reduction in overall mortality in current and former smokers who are at high risk. The USPSTF recommends annual LC screening with an LDCT scan for adults aged 50-80 years who either are currently smoking with a 20-pack-year smoking history or have quit within the past 15 years. However, there is a lack of clarity regarding small, indeterminate nodules that are monitored by repeated CT scans or biopsies. AI models can better identify nodules that require investigation and prevent unnecessary anxiety-provoking interventions [73]. Nodule detection using CAD using multiview convolutional networks has very high sensitivity (90%) and high detection of actionable nodules. Some examples are the AI-RAD Companion (AUC = 0.942) and DL CADe systems (AUC = -0.989). AI-based radiomics models, such as SVM-LASSO (AUC = 0.89), classify nodules with a high degree of accuracy. Segmentation of nodules was performed with a high degree of accuracy using specific AI models, such as SD-Unet. Analysis of various components such as autoantibodies, complement fragments, miRNA, tumor DNA, and serum proteins as potential screening biomarkers for LC using ANNs has increased LC prediction rates from 85% to 87.3%. Image analysis using AI models like DFCNet or CNN had an overall accuracy of 84.58% and 77.6%, respectively [61].

Many incidentally detected nodules are benign granulomas, and up to 12% of nodules are malignant. Current practice guidelines suggest follow-up with repeated imaging for three to 13 months and using radiologic features to score these nodules manually. Although this can be cumbersome, it also leads to considerable inter-reader variability and biases, which can be minimized using an AI-integrated approach.

The management approach of observation vs. biopsy is dictated by radiologic features, which are not based on accurate decision-making tools and can potentially result in either delaying the treatment of a true positive case or leading to increased mortality and morbidity from erroneous invasive testing of true negative patients. AI could emerge as a useful clinical tool that can be used to identify and predict the risk of future cancer incidence and to differentiate between indolent nonmalignant nodules and malignant and aggressive cancers [74].

In the immunohistochemical analysis of tumors, ConvPath, one CNN model, achieved an overall accuracy of 90.1%. Further research using AI in liquid biopsy sample analysis is ongoing. Cook et al. demonstrated utilizing a subset of ML called unsupervised learning to identify novel mutations in lung adenocarcinoma and squamous cell carcinoma, such as the PGX (an oncogenic driver in breast cancer) mutation [22].

AI has also been integrated into drug discovery and development at the transcriptomic level to investigate chemical structures. Pimozide, an anti-dyskinesia agent, has been found to be a useful candidate in the management of NSCLC in this manner. Investigations by Charoentong et al. [70] on PDL-1 and Kureshi et al. [71] on EGFR in NSCLC have shown the utility of AI in optimizing immunotherapy in LC. Optimization of radiotherapy using AI-based models has been demonstrated by Ladbury et al. using AAR-RT, which identifies organs at risk from CT images and tailors radiation regimens, which makes treatment faster and more efficient [22]. Similarly, models like QUANIC are used to monitor responses and resistance to immunotherapy [66].

AI offers better surgical risk stratification for LC patients, including no surgery if deemed high-risk. They used NN (AUC = -0.98) to predict postoperative cardiorespiratory morbidity.

AI is utilized by robotic surgery systems like smart tissue autonomous robots to optimize and automate complex surgery planning [22]. AI systems, such as WFO, have been used to predict surgical risk in LC patients. AI systems are also being utilized at various stages of drug development [66].

Comparative Analysis of AI-Assisted Approaches

LC is associated with a late diagnosis at an advanced stage, resulting in a high mortality rate. Simultaneously, we need to look beyond AI by integrating clinical data, genomics, radiomics, and semantic models to build the best model. The large body of existing data comparing the utility of the ML and DL models emphasizes this fact.

Radiomics-based AI techniques are as effective as or potentially superior to human expertise in tumor detection, histology, staging, treatment response assessment, prognosis prediction, and prediction of recurrence and metastasis, although FDG PET/CT remains vital for NSCLC diagnosis and staging. Differentiating between benign and malignant nodules pathologically and radiologically has been investigated by Wang et al. using deep learning techniques such as deep LN and CNN, respectively [63]. CNNs have also been demonstrated to be effective in the segmentation of lung lesions in medical images using the RAD-UNet AI model. A newer AI-based histogram technique to assess lung nodules was investigated by Agarwal and Guo [75].

Ren et al. aim to develop a clinical-biological-radiomics model via ML for assessing lymph nodes in LC patients [76].

Limitations in Real-World Applications

AI relies heavily on data, and data extraction from EMR remains a challenge. Acquiring a large sample size to train, test, and validate AI models must also be a challenge. This can affect the generalizability of AI research [22]. Although publicly available datasets like LIDC-IDRI and LUNA 16 exist, collaboration between multiple institutions worldwide is a better way to improve the generalizability and replicability of the generated results [61].

The lack of reporting standards also affects the reproducibility of AI research models. However, these studies require external validation to reduce inconsistencies and errors. Although there have been studies generating hypotheses, very few studies have compared AI-based interventions to the standard of care, and this needs to be worked on. The wider availability of data through establishing central repositories can increase the reproducibility of data [22].

At the human level, the lack of resources, infrastructure, and training among healthcare professionals hinders the application of AI tools in day-to-day clinical practice, and these need to be addressed at the healthcare system level. AI tools need to be seamlessly integrated into the EMR and updated regularly to suit the changing healthcare needs of the population and as per the feedback from clinicians. Guidelines ensuring patient safety, privacy, and data security in compliance with HIPAA regulations must be established before using AI systems in clinical practice [61]. Finally, cognitive bias emerging from a lack of trust and understandability despite demonstrated efficacy poses a serious challenge to implementing AI models in patient care [22]. This needs to be addressed by providing a rationale behind the decisions made using AI-based software, enhancing physicians’ trust, and promoting the principles of SDM with patients [61].

Patient outcomes and quality of care

While AI tends to have an impact on diagnosis and management, it is important to quantify just how much of an impact it has on treatment responses and survival rates to determine whether it is both feasible and whether it can replace the current diagnostic algorithms. Recent studies have shown that LC can be detected at 94% AUC, with it outperforming radiologists by absolute reductions of 11% in false positives and 5% in false negatives when prior CT scans were not available and being on par when these images were available [42]. Another study where a commercially available CAD was used demonstrated higher actionable data for AI-detected lung nodules than non-AI-detected lung nodules [77].

The significance of the detection rate and its improvement begs the question of whether AI can be safely and ethically implemented. Four major ethical issues have been constantly brought up with regard to ethical issues: (1) informed consent to use data; (2) safety and transparency; (3) algorithmic fairness and biases; and (4) data privacy are all important factors to consider [78]. If we are able to solve a majority of these issues, there still lies a deeper moral question on who ultimately takes culpability for the outcomes of the AI algorithm, and even if we are able to pinpoint culpability based on the sheer possibility of AI learning new patterns and deviating from its norm, operating without a framework would ultimately mean that culpability is redundant [79], and without a legal framework that solves all these issues, AI could be potentially more dangerous than useful.

Assuming we achieve the utopia of AI algorithms both diagnosing and deciding treatment patterns for patients, ultimately patients would be willing to be treated knowing that their treatment was being determined by an algorithm. Certain studies have shown mild acceptability for AI health chatbots, with people with poorer IT skills being less comfortable with the idea of using these AI chatbots [80].

In one of the largest studies published in The Lancet, AI acceptability was analyzed in detail, which showed overall general acceptability in many regions of healthcare, but the results were heavily skewed, with about 20% of people completely opposed to any biomedical device or use of AI in their healthcare plans, with trust being the main sticking point, and an interesting point that was noted was that there were no population-related measures that were linked to AI acceptability [80]. Ultimately, patient independence is paramount, and all decisions must be informed and determined keeping the patients’ best interests in mind.

Future directions and challenges

Emerging Technologies in AI for LC

Beyond the current CT image-based learning models for diagnosing LC, a multitude of emerging AI techniques have shown promise. These encompass a broad spectrum of data sources, including EHRs, imaging modalities, histopathology, and molecular biomarkers, all aimed at enhancing the accuracy of disease risk prediction, diagnosis, and prognosis of treatment response [3].

Some of the burgeoning AI models include federated learning models, multimodal deep learning models amalgamating data from diverse modalities, and interpretable deep learning models that fuse neural networks with domain knowledge from radiologists [4]. Ongoing efforts focus on refining approaches to classify identified nodules more accurately and reduce false-positive rates.

CNNs stand out as one of the most interpretable AI algorithms for image analysis and classification [3]. Employing CNNs, analysts aim to discern visual patterns by subjecting raw pixel data to a series of transformations and filters [1]. CNNs are instrumental in scrutinizing various lung nodule features such as morphology, shape, and growth rate post-detection [2].

Another burgeoning field is radiomics, which leverages medical imaging to generate high-dimensional quantitative data that underpins comprehensive diagnosis and patient care. Radiomics also bolsters CADe by integrating radiologic findings with radiomic features for enhanced tumor characterization. The Computer-Aided Nodule Assessment and Risk Yield tool exemplifies such an approach, demonstrating promise in predicting a subset of lung adenocarcinoma patients [1].

Potential Barriers to Widespread Adoption

Despite the promise of AI, several potential barriers have hindered its widespread adoption. Primarily, reliance on public databases like the LIDC-IDRI introduces sampling bias, impacting the validity of AI models [3].

Challenges in data acquisition and organization persist, as large sample sizes are essential for validating diverse AI models. Current outcomes-based research studies often involve relatively small patient cohorts, necessitating thousands of participants for robust applications [1].

Ethical concerns loom large in AI adoption, particularly regarding patient confidentiality and autonomy. Obtaining patient consent becomes crucial, especially given the potential consequences of erroneous AI predictions [1].

Moreover, most medical AI tools currently focus on detecting single diseases, complicating the identification and distinction of various ailments.

Areas for Further Research and Development

Addressing these barriers necessitates concerted research and development. Initiatives aimed at increasing sample size and data availability, such as the Cancer Imaging Archive and The Cancer Genome Atlas, are crucial [1].

To optimize the screening process, future research should integrate AI technologies in a distinctive manner. This includes prescreening applications for customized risk assessment, the acquisition of images using low-dose protocols, reconstruction via deep learning algorithms to preserve image quality with minimal radiation exposure, and AI-based systems for automated nodule detection to alleviate the radiologist’s burden. The classification of nodules as benign or malignant represents a critical step in resource management and avoiding unnecessary procedures [2].

Furthermore, exploring the potential of AI in examining dietary, lifestyle, and behavioral patterns for early identification of LC [15]. Interdisciplinary collaborations and continued innovation will be essential to overcome these challenges and harness the full potential of AI in LC management.

Future Implications and Directions

Looking ahead, the integration of AI technologies into LC management protocols for smokers heralds a shift in treatment paradigms and patient outcomes. Continuous refinement of AI algorithms is paramount, necessitating interdisciplinary collaborations between AI experts, oncologists, public health officials, and policymakers [2]. Through initiatives such as the Cancer Imaging Archive and The Cancer Genome Atlas. Efforts to augment data availability and sample sizes will propel AI research forward [1]. Future research priorities include optimizing AI-driven screening processes, integrating AI into preventive measures tailored to individual risk profiles, and leveraging AI to address disparities in access to early detection and personalized treatment [15]. Ultimately, the long-term impact of AI integration lies in its potential to democratize healthcare, enhance treatment efficacy, and improve overall survival rates among smokers with LC.

**Figure 3:**
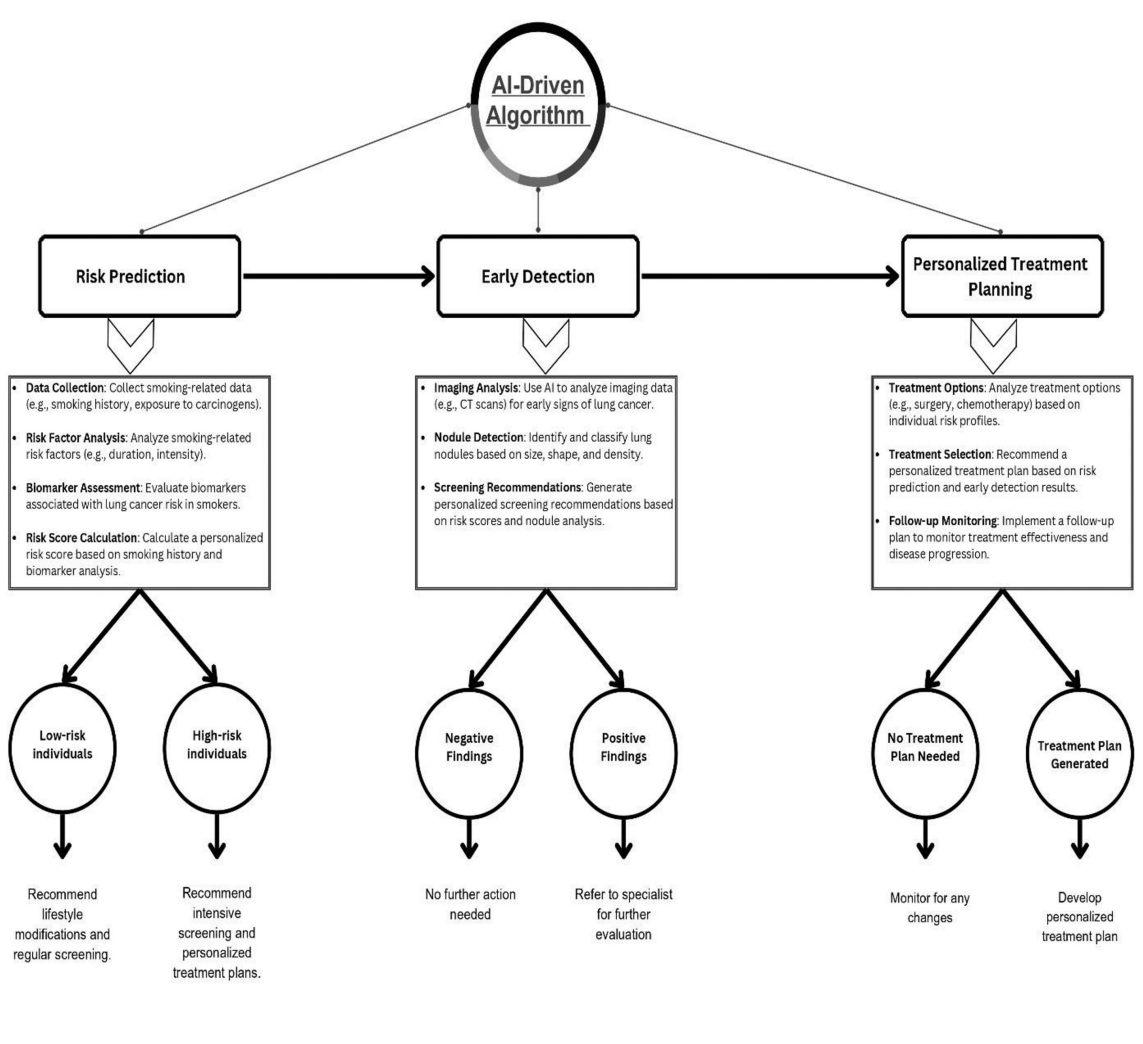
Algorithm illustrating an AI-driven approach as a beacon of innovation and hope for smokers AI, artificial intelligence

In envisioning a future in which AI plays a pivotal role in combating LC among smokers, our proposed algorithm (Figure 3) stands as a beacon of innovation and hope. By harnessing the power of ML models, this algorithm revolutionizes risk prediction, early detection, and personalized treatment planning tailored specifically for smokers, a group disproportionately affected by this disease.

Our algorithm builds upon the success of existing AI systems in detecting the early stages of LC, often with higher accuracy than trained radiologists. For instance, a joint initiative between Google, Northwestern University, and other institutions has shown promising results in detecting the early stages of LC [81]. Studies have indicated that AI algorithms, when assisting radiologists, significantly improve their performance in detecting LC on chest X-rays. In a meta-analysis, AI-aided diagnosis systems demonstrated a combined sensitivity of 0.87 and specificity of 0.87 for LC diagnosis, with a missed diagnosis rate of 13% and a misdiagnosis rate of 13% [82-84].

What sets our algorithm apart is its adaptability and continuous refinement based on emerging research findings and advancements in LC treatment. By integrating new data and updating its algorithms, this system ensures that patients receive the most cutting-edge and effective care available, thereby enhancing treatment outcomes and survival rates. For example, ML architectures have demonstrated promising results in detecting and classifying LC across different lesion types, with sensitivities ranging from 0.81 to 0.99 and specificities from 0.46 to 1.00. The accuracy of these ML algorithms has ranged from 77.8% to 100% [85].

Moreover, the algorithm’s emphasis on interdisciplinary collaboration between AI experts, oncologists, public health officials, and policymakers is pivotal. This collaboration not only facilitates the implementation and dissemination of AI-driven interventions for LC prevention and treatment among smokers but also ensures that these interventions are ethical, accessible, and equitable.

The integration of AI technologies into LC care using this algorithm offers a beacon of hope. This has the potential to significantly reduce mortality rates, improve patient outcomes, and enhance the quality of life of smokers with this devastating disease.

## Conclusions

The comprehensive review addresses the vital significance of AI in developing the treatment of LC. With smoking as a significant risk factor, the need for innovative strategies to enhance early detection and personalized treatment is critical. AI-powered technologies, leveraging ML and advanced imaging, promise to augment traditional approaches, enabling accurate risk identification and personalized treatment plans. The integration of AI in LC care has the potential to revolutionize diagnosis and therapy, improving patient outcomes. However, realizing this potential requires substantial investment in robust data infrastructure and collaborative efforts across the healthcare ecosystem. Prioritizing ethical factors, such as maintaining patient privacy and ensuring fair access, is of utmost importance. Ultimately, a collective commitment to innovation, collaboration, and ethical stewardship is essential for fully harnessing the potential of AI in LC care, ultimately aiming to reduce mortality rates and enhance the quality of life for those affected by this devastating disease.
